# Drug Combination of Ciprofloxacin and Polymyxin B for the Treatment of Multidrug–Resistant *Acinetobacter baumannii* Infections: A Drug Pair Limiting the Development of Resistance

**DOI:** 10.3390/pharmaceutics15030720

**Published:** 2023-02-21

**Authors:** Junwei Wang, Marc Stegger, Arshnee Moodley, Mingshi Yang

**Affiliations:** 1 Department of Pharmacy, Faculty of Health and Medical Sciences, University of Copenhagen, Universitetsparken 2, DK-2100 Copenhagen, Denmark; 2 Department of Bacteria, Parasites and Fungi, Statens Serum Institut, DK-2300 Copenhagen, Denmark; 3 Department of Veterinary and Animal Sciences, University of Copenhagen, DK-1870 Frederiksberg C, Denmark; 4 Animal and Human Health, International Livestock Research Institute, Nairobi 00100, Kenya; 5 Wuya College of Innovation, Shenyang Pharmaceutical University, Wenhua Road No. 103, Shenyang 110016, China

**Keywords:** drug combination, inhalable dry powders, resistance development, synergistic effect

## Abstract

Polymyxins are considered as last–resort antibiotics to treat infections caused by *Acinetobacter baumannii*. However, there are increasing reports of resistance in *A. baumannii* to polymyxins. In this study, inhalable combinational dry powders consisting of ciprofloxacin (CIP) and polymyxin B (PMB) were prepared by spray–drying. The obtained powders were characterized with respect to the particle properties, solid state, in vitro dissolution and in vitro aerosol performance. The antibacterial effect of the combination dry powders against multidrug–resistant *A. baumannii* was assessed in a time–kill study. Mutants from the time–kill study were further investigated by population analysis profiling, minimum inhibitory concentration testing, and genomic comparisons. Inhalable dry powders consisting of CIP, PMB and their combination showed a fine particle fraction above 30%, an index of robust aerosol performance of inhaled dry powder formulations in the literature. The combination of CIP and PMB exhibited a synergistic antibacterial effect against *A. baumannii* and suppressed the development of CIP and PMB resistance. Genome analyses revealed only a few genetic differences of 3–6 SNPs between mutants and the progenitor isolate. This study suggests that inhalable spray–dried powders composed of the combination of CIP and PMB is promising for the treatment of respiratory infections caused by *A. baumannii*, and this combination can enhance the killing efficiency and suppress the development of drug resistance.

## 1. Introduction

Respiratory infections are a leading health threat causing millions of deaths worldwide annually [1]. Moreover, the number of respiratory infections caused by multidrug–resistant (MDR) bacteria is growing rapidly, especially those infections caused by *Pseudomonas aeruginosa* [2], *Klebsiella pneumonia* [3] and *Acinetobacter baumannii* [4], and are they associated with high morbidity and mortality [5].

Among those bacterial species, *A. baumannii* should be highlighted as it is extremely difficult to treat and can readily acquire resistance to multiple antibiotics during treatment [6]. In addition, this pathogen usually survives in hospital environment and can cause nosocomial infections, especially in patients in intensive care units (ICUs). Polymyxins such as polymyxin B and polymyxin E (also known as colistin) are considered as the last–resort treatment of *A. baumannii* infections, since other antibiotics are less effective [7]. However, there are increasing reports of unsuccessful polymyxin monotherapy of *A. baumannii* respiratory infections [8], and hence polymyxin resistance is becoming an inevitable therapeutic issue [9,10,11].

Combination therapy has been shown to contribute to better clinical outcomes than monotherapy, improving the survival of patients suffering from MDR pathogens infections [12,13,14], especially when a combination exhibits synergistic effect. Drug pairs with synergistic effects can afford an amplified antibacterial effect compared to single used antibiotics, enhancing bacteria kill rate and narrowing the time window of the resistant development [13]. A polymyxin–based combination treatment has been considered as a promising treatment option against MDR *A. baumannii* [15,16,17]. In a clinical study, a synergistic effect was observed when polymyxin B was combined with levofloxacin, tobramycin and meropenem to treat MDR *A. baumannii* infections [18]. However, the antibacterial effect will also eliminate susceptible bacteria, and antibiotic residues can select for mutants with a reduced susceptibility to the same antibiotics [19,20]. Furthermore, the wide use of drug combinations can also result in cross–resistance or collateral sensitivity [20]. Nevertheless, it is promising that recent studies have shown that cross–resistance can be used to rationally design dosing regimens to avoid resistance development instead of promoting it [21,22]. Therefore, it is important to assess not only the killing efficiency but also the drug resistance development when adopting antibiotic combination therapy [23,24].

The combination of polymyxins and fluoroquinolones has been reported to be effective against MDR *P. aeruginosa* and MDR *A. baumannii* [25,26], but the re–sensitization of the strain by this antibiotic combination has not been reported. In this study, a combination of ciprofloxacin (CIP) and polymyxin B sulfate (PMB) was formulated into inhalable dry powders and tested against *A. baumannii* strain K31. The dry powders were characterized and evaluated with respect to the particle properties, solid state, in vitro aerosol performance, in vitro dissolution and in vitro antibacterial effect. In addition, the antibacterial activity and resistance evolution of the PMB and CIP combination were investigated.

## 2. Materials and Methods

### 2.1. Chemicals

United States Pharmacopeia (USP) standard CIP and PMB were purchased from Nanjing Sunlida Biological Technology Co., Ltd. (Nanjing, China). Sodium sulfate and acetonitrile were purchased from Sigma–Aldrich (Copenhagen, Denmark); dialysis bags (Biotech RC Dialysis Tubing) with 20 kD typical molecular weight cut–offs were obtained from Spectrum Laboratories (Compton, CA, USA). E–TEST strips (CIP and PMB) were purchased from bioMérieux SA (Marcy–l’Étoile, France).

### 2.2. Strains

*A. baumannii* K31 is a human clinical strain that was isolated in August 2017 from a wound infection (part of the biorepository at Department of Veterinary and Animal Sciences, University of Copenhagen). Minimum inhibitory concentrations (MICs) to aztreonam, CIP, PMB and tobramycin are presented in Table 1.

### 2.3. Wet Milling

As a hydrophobic compound, ciprofloxacin was prepared by media milling methods to form a homogeneous nanocrystal suspension to be used as feeding solutions [28]. Briefly, 1.0 g of raw ciprofloxacin was dispersed in 20 mL Poloxamer 188 (F68) aqueous solution (0.5%, *w*/*v*). Glass beads with two different sizes (1 mm and 2 mm diameter) were mixed as media in the milling process. This mixture was then homogenized for 24 h at room temperature with ~40 mL of milling media by a magnetic stirrer (900 rpm) to prepare nanocrystal suspensions. The micronized particles were collected by centrifugation, washed twice with purified water and dispersed again as feeding solution.

### 2.4. The Spray–Drying Process

CIP and PMB dry powders were prepared using a Büchi 290 spray drier (BÜCHI Labortechnik; Falwil, Switzerland). The susceptibility profiles (MICs) of antibiotics were important as a preliminary guidance for formulation preparation. In this study, a ratio of 32:1 (CIP/PMB) was determined to be used in the combination dry powder. This ratio was selected based on the MIC value of CIP and PMB. Feeding solutions for each formulation and corresponding composition are listed in Table 2. PMB was dissolved in water or CIP suspension to be prepared as the feeding solutions. The spray–drying conditions were as follows: the inlet and outlet temperature were 100 °C and 46–52 °C respectively; the drying airflow rate was 35 m^3^/h; the atomization air flow rate was 700 L/h; the feeding rate of the solution/suspension was 3 mL/min. The spray–dried powders were collected in glass vials and stored in a desiccator at room temperature until further characterization.

### 2.5. Morphology

Samples were fixed on a sticky carbon tape and sputtered with gold by a sputter–coater (Leica EM ACE200, Leica Microsystems GmbH, Wetzlar, Germany). Images were captured at an acceleration voltage of 2.00 kV by scanning electron microscopy (SEM) (Quanta 3D FEG, Thermo Fisher Scientific, Waltham, MA, USA).

### 2.6. Particle Size

The mean particle sizes of the dry powders were determined by laser diffraction (Malvern Mastersizer 2000, Malvern Instruments, Malvern, Cambridge, UK) equipped with a dry powder feeder unit (Scirrocco 2000 powder feeder, Malvern Instruments Malvern, Cambridge, UK). Dry powder samples were dispersed by air at a pressure of 3 bars. The refractive index was set to 1.520 for the measurement of the samples. The samples were measured in triplicate. The size distributions of the samples are presented via the span, calculated using the following equation:

(1) $$Span=\frac{{Dv}_{90}-{Dv}_{10}}{{Dv}_{50}}$$

The Dv_10_, Dv_50_, and Dv_90_ were also reported, which refer to the volumetric diameter at 10%, 50% and 90% cumulative number, respectively.

### 2.7. X-ray Powder Diffraction (XRPD)

The XRPD patterns of the powders were collect by an X-ray diffractometer (X’Pert PRO MPD, PANalytical, Almelo, The Netherlands) with a slit detector Ni–filtered CuKα1 source generated at 40 mA and 45 kV. Data were collected from 5° to 38° 2θ, the step width was 0.04° and the scan speed was 5°·min^−1^. The diffraction patterns of unprocessed raw materials (i.e., CIP, and PMB), physical mixtures of raw materials and spray–dried samples (i.e., CIP–SD, PMB–SD, PMB–CIP–SD) were collected.

### 2.8. Dynamic Vapor Sorption (DVS)

The water sorption–desorption properties of the samples were described by a VTI–SA^+^ (TA Instruments, New Castle, DE, USA). Sample preparation began with a drying step then continued with a sorption–desorption cycle. Briefly, approximately 10 mg of powder for each sample was added in a quartz holder, and then exposed in the instrument under 0% relative humidity (RH) at 60 °C for 180 min or until a constant weight (less than 0.001 wt. % change over 5 min) was reached. After drying, samples were cooled down and maintained at 25 °C. The samples were exposed to the following sorption–desorption cycle: 0 to 90% in 10% step size and the same for desorption. Each step’s running time was less than 120 min, or until it reached an equilibrated weight (less than 0.001 wt. % change) over 5 min. Data were recorded every 2 min or when a ≥0.0100 wt. % change occurred. Profiles of weight records during the sorption–desorption cycle were collected to present the water sorption–desorption properties of the samples.

### 2.9. In Vitro Dissolution

Specific amounts (200 mg approximately) of CIP–SD, PMB–SD, PMB–CIP–SD and raw materials (CIP and PMB) were added to dialysis bags and sealed individually. Each dialysis bag was then transferred into 200 mL of dissolution medium (50 mM PBS, pH 7.4). All samples were incubated in a shaking water bath (100 rpm) at 37 °C. At predetermined time points (20 min, 40 min, 1 h, 1.5 h, 2 h, 4 h, 8 h and 24 h), 10 mL of dissolution medium was withdrawn and replaced with fresh medium. Samples were centrifuged first. The concentrations of CIP and PMB were measured using an HPLC (1260 Infinity, Agilent Technologies, Santa Clara, CA, USA) with a reverse–phase column (Agilent C18 150 × 4.6 mm, Agilent Technologies, USA). The mobile phases consisted of a 26% acetonitrile and 74% 30 mM solution of sodium sulfate (pH 2.5, adjusted with phosphoric acid), and the flow rate was 1 mL/min. The samples were detected at 215 nm by a UV detector. Calibration curves were prepared for CIP (1–40 µg/mL, limit of quantification was 50 ng/mL, r^2^ > 0.999) and PMB (10–100 µg/mL, limit of quantification was 8 µg/mL, r^2^ > 0.999). The peak areas for polymyxin B1 and B2 were summed for the quantification of PMB. The dissolution rates of the samples were compared via their cumulative dissolution profiles. All samples in the dissolution study were tested in triplicates.

### 2.10. In Vitro Aerosol Performance

The in vitro aerosol performances of the spray–dried powders were assessed using a Next Generation Impactor (NGI, Copley Scientific, Nottingham, UK). Prior to the tests, the collection plates of NGI were coated with a Tween 20 solution (0.5% (*w*/*v*)) to minimize particle bouncing. A low–resistance–type RS01 Monodose dry powder inhaler (Plastiape, Osnago, Italy) was used as the dry powder inhaler device for all tests, and a mouthpiece adapter was used to connect the inhaler to the throat (stainless steel USP induction) of the NGI. About 30 mg of dry powder was put into size 3 hydroxypropyl methylcellulose (HPMC) capsules (Capsugel, Greenwood, SC, USA) for each formulation. One capsule was loaded in the inhaler and emitted in each run at an air flow rate of 90 L/min for 2.6 s. The corresponding pressure drop for the device was adjusted to ~3.9 KPa with the current NGI setting. The powders deposited in the stages of the NGI, the USP throat, the capsule and the inhaler were collected with 1.7% (*v*/*v*) acetic acid solution, and the concentrations of CIP and PMB in the samples were determined by HPLC (described in the Section 2.9). Fine particle fractions (FPF) and emitted dose (ED) were calculated for the evaluation of aerosol performance. The FPFs of the formulations were calculated as the percentage mass of the drug with an aerodynamic diameter smaller than 5 µm of the drug collected from the tests. ED values were defined as the mass percentage of drug recovered from all NGI parts relative to the total drug recovered from the experiments. For each formulation, three independent batches were used for the in vitro aerosol performance evaluation.

### 2.11. Time–Kill Assay

Overnight bacterial cultures of *A. baumannii* K31 were adjusted to 5 × 10^5^ colony–forming units (CFU)/mL in cation–adjusted Mueller–Hinton broth (200 mL), transferred to glass flasks and incubated for 30 min at 37 °C to reach the early exponential growth phase. Spray–dried powders (CIP–SD, PMB–SD and PMB–CIP–SD) were sealed in a dialysis bag and added to each culture flask at the time point of zero. A sequential combination group was set here to compare with the fixed–dose combination. For this sequential addition, PMB–SD was added at time point zero with other groups, and the CIP–SD was added after sampling at 24 h. The experimental design and treatment groups are described in Table 3. After the antibiotics were added, the flasks were incubated at 37 °C for 48 h. At time intervals of 2 h, 6 h, 12 h, 24 h, 36 h and 48 h, 150 µL of culture was withdrawn from the flasks and serially diluted in saline. The diluted culture was then spotted (20 μL of each dilution) on Mueller–Hinton agar plates. After 24 h incubation, the colonies were counted, and CFU/mL values were calculated for each group. Experiments were performed in duplicate, and data have been presented as the mean value of the two counts. To verify the dissolution influence when using drug powders in the time–kill study, a parallel study was performed by employing the antibiotic solution with the same group setting as that used in the spray–dried powders group (description in Supplementary Results).

### 2.12. Population Analysis Profiling (PAP)

**Figure 1:**
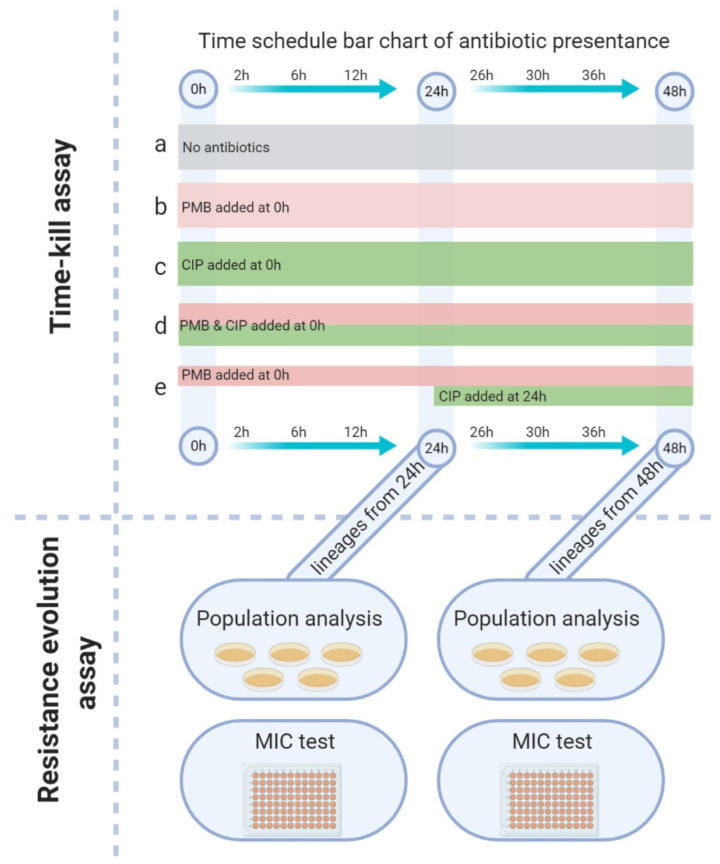
Graphic representation of the in vitro microbiology study. Colored bars in the time schedule represent different addition times and durations of spray–dried antibiotic dry powder application; the group codes correspond to Table 3. Lineages from different time–kill assay (at 24 and 48 h) groups were used for further assaying resistance development.

Bacteria from the different treatment groups in the time–kill assay were included in the PAP analysis (Figure 1). Briefly, at time intervals of 24 h and 48 h, bacteria were sampled from each group of the time–kill assay, centrifuged and washed twice, and then spread on a 5% blood agar plate and incubated at 37 °C overnight. After incubation, three colonies from each of the 24 h and 48 h plates were randomly selected, and each strain yielded a lineage that was then used for PAP analysis and confirmatory MIC testing. Antibiotic–free solutions were used as a reference.

Each isolate was adjusted to 0.5 MacFarland (10^8^ CFU/mL) in 0.85% saline. The starting suspension and serial dilutions (10–1 to1–6 diluted in 0.85% saline) were spotted (20 µL) on Mueller–Hinton agar plates without or with varying concentrations of PMB (0.5, 1, 2, 4, 8, and 16 µg/mL) and CIP (16, 32, 64, 128, 256, and 512 µg/mL). Colonies were counted after 48 h of incubation at 37 °C. PAP values were based on CFU counting and the corresponding drug and drug concentration colonies were counted. The results are grouped with the corresponding group in the time–kill assay (24 h and 48 h).

### 2.13. MIC Testing

The MICs of all isolates at the start of the PAP experiment were determined by E–TEST for CIP and PMB, performed according to the manufacturer’s recommendations (BioMérieux, Marcy–l’Étoile, France). Briefly, each isolate was adjusted to 5 × 10^8^ CFU/mL and spread using a cotton swab on a Mueller–Hinton agar plate. E–test strips were placed on the plates and incubated at 37 °C for 20 h, and the MIC was read where the bacterial growth intersected the test strip. Isolates with MICs below test limits were re–tested by broth microdilution method. Briefly, two–fold serial dilutions from 1 to 512 µg/mL were diluted in Mueller–Hinton broth in a 96–well microtiter plate, and a 0.5 MacFarland standard inoculum (fresh overnight culture) of each group was diluted and transferred into each well to afford a final inoculum of 5 × 10^5^ CFU/mL. All plates were incubated for 20 h at 37 °C, and the MIC value was the concentration at which no bacterial growth was visible in the well. The MICs of the isolates were compared with the original *A. baumannii* K31 (Table 1), and value changes were recorded. The results have been grouped via the corresponding group name in the time–kill assay (24 h and 48 h). For each lineage in the time–kill groups, three isolates were selected, and data have been presented as the mean and standard deviation.

### 2.14. Genome Sequencing and Analysis

The 48 h lineage values after time–kill study were used for the identification of sequence variations. The lineages derived from solutions were used to avoid the uncertainty of dry powders (Supplementary materials, Table S2). From the original K31 strain, as well as the 48 h lineages of the control, PMB–Sol and CIP–Sol groups, one colony was picked for genome sequencing analysis. For the 48 h lineages of the PMB–CIP–Sol and PMB–Sol–CIP–Sol groups, three colonies were picked for sequencing.

All colonies were then re–cultured overnight in Mueller–Hinton broth at 37 °C. Genomic DNA was extracted using the DNeasy Blood and Tissue kit (Qiagen, Venlo, The Netherlands). The purity and concentration of extracted DNA were assayed using the Nanodrop and Qubit instruments, respectively. The Nextera XT library preparation kit was used to prepare a sequencing library. The prepared library was sequenced on a MiSeq using a paired–end 2 × 250 bp sequencing strategy, according to standard Illumina protocols (Illumina, San Diego, CA, USA).

To identify sequence variations, the raw sequence data were aligned to an annotated *A. baumannii* 1656–2 reference chromosome (GenBank accession no. CP001921) [29] using NASP v1.2.0 [30] by using BWA–MEM v0.7.12 [31], and the variants were called using GATK [32]. To retain only high–quality variant calling, the respective position was not included when a minimum of 10–depth sequencing was not met, or the nucleotide variant was shown in <90% of the base calls per individual isolates.

### 2.15. Statistical Analysis

The results are indicated with the appropriate number of replicates (n) and represented as the mean value ± standard. Statistics were carried out using GraphPad Prism version 8.0 for Windows. *p*-values below 5% (*p* < 0.05) were considered as statistically significant, as determined by analysis of variance (ANOVA) followed by a *t*-test.

## 3. Results

### 3.1. Preparation and Characterization of Spray–Dried Powders

**Figure 2:**
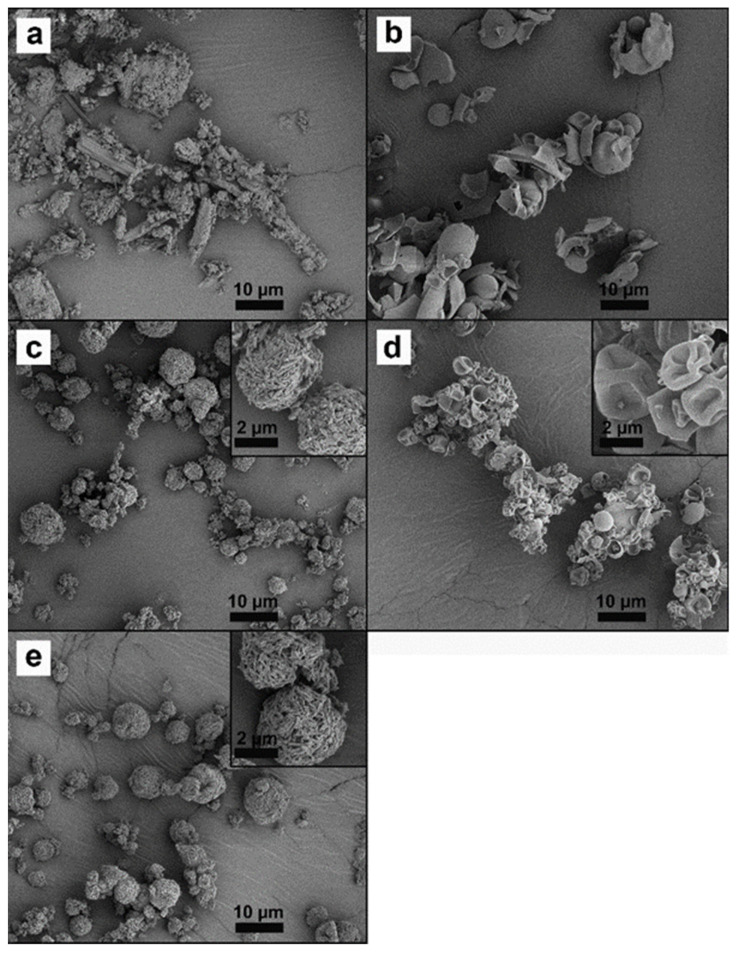
SEM pictures of raw CIP (**a**), raw PMB (**b**), CIP–SD (**c**), PMB–SD (**d**) and PMB–CIP–SD (**e**).

Prior to spray–drying, the CIP was wet–milled to nanoparticles of around 362 nm, as measured by dynamic light scattering (description in Supplementary results). Upon spray–drying, the CIP nanoparticles obtained from the wet–milling were transformed to spherical particles, i.e., CIP–SD, in a size range of 1–6 µm (Table 4), measured by laser diffraction. The CIP–SD particles were spherical and composed of fine grains (Figure 2c). The spray–dried PMB (PMB–SD) particles were hollow and wrinkled, in a size range of 0.7–6.4 µm (Table 2). Co–spray–dried samples, i.e., PMB–CIP SD, resembled CIP–SD particles (Figure 2e), and their sizes ranged 0.7–6.4 µm (Table 2).

**Figure 3:**
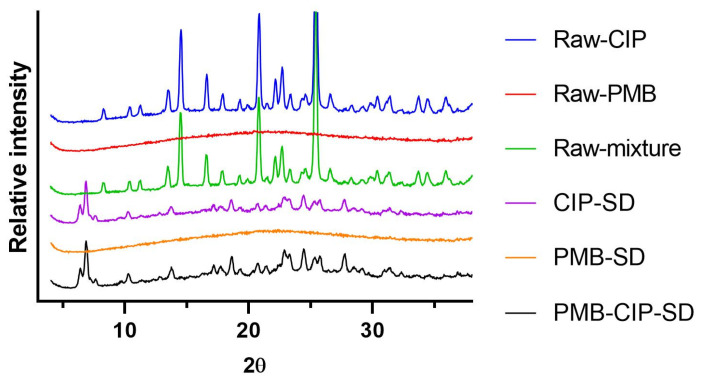
Powder diffraction patterns of raw CIP, raw PMB, physical mixture, CIP–SD, PMB–SD and PMB–CIP–SD.

As for the solid states of the different formulations, the PMB remained amorphous after the spray–drying process, whereas the CIP–SD exhibited different crystalline forms from raw CIP (Figure 3). The diffraction patterns of the co–spray–dried PMD–CIP–SD powders resemble those of CIP–SD.

**Figure 4:**
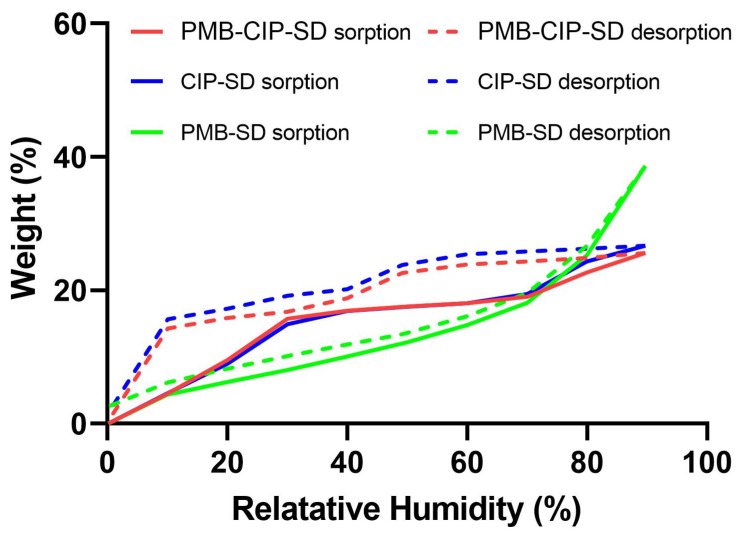
Dynamic vapor sorption isotherm of different formulations. The solid line and dotted line represent the sorption and desorption processes, respectively.

In the DVS analyses, CIP–SD exhibited a rapid water sorption until 30% RH, followed by a slow water sorption in the range of 30–70% RH, and another burst of rapid water sorption until 90% RH (Figure 4). The CIP–SD underwent a total of 25% (*w*/*w*) of its weight gain up to 90% RH. The removal of water from the CIP–SD seemed to complete with desorption, whereas the desorption exhibited a different profile from the sorption. There was negligible desorption of water from 90% to 50% RH, which was followed by a rapid loss of water from 50% to 40% RH, and from 20% to 10% RH. In contrast, the sorption and desorption profiles of PMB–SD are mostly overlaid, with a total 38% (*w*/*w*) of the weight gain occurring up to 90% RH. The sorption–desorption profiles of PMB–CIP–SD resemble those of CIP–SD.

### 3.2. In Vitro Dissolution and Aerosol Performance of Spray–Dried Powders

**Figure 5:**
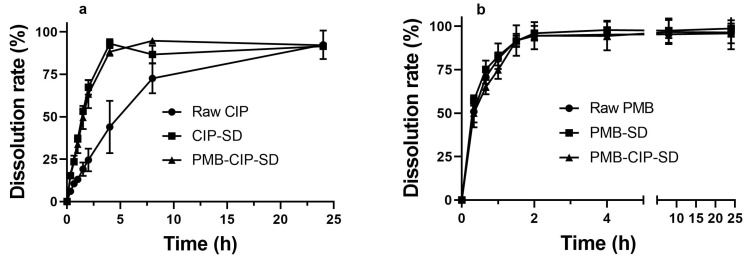
In vitro accumulated dissolution profiles of CIP (**a**) and PMB (**b**) (mean ± SD, n = 3).

The dissolution rates of PMB from different samples, i.e., raw PMB, PMB–SD and PMB–CIP–SD, are similar, and they were all faster than CIP (Figure 5). The dissolution rates of spray–dried CIP samples i.e., CIP–SD and PMB–CIP–SD, were similar, and were faster than that of raw CIP.

**Figure 6:**
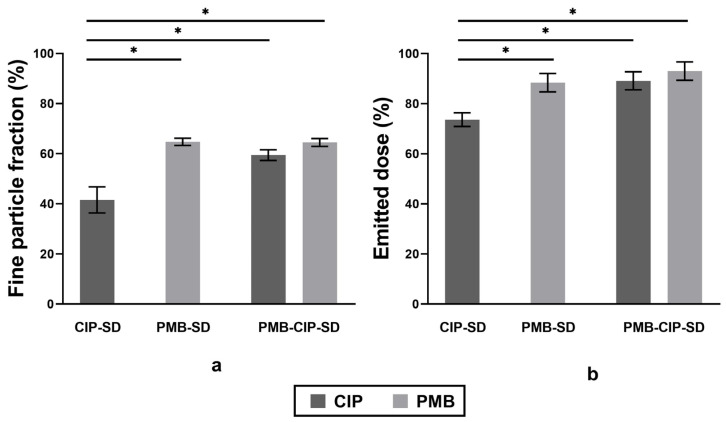
Fine particle fractions (FPF) (**a**) and emitted dose (ED) (**b**) of spray–dried formulations (mean ± SD, n = 3). Significant differences (*p* < 0.05) are indicated with asterisk.

All spray–dried powders exhibited relatively high FPF and ED values of over 40% and 70%, respectively (Figure 6). PMB–SD exhibited significantly higher FPF and ED compared to CIP–SD. The FPF and ED values of PMB–CIP SD are similar to those of PMB–SD (*p* < 0.05).

### 3.3. Time–Kill Assay

**Figure 7:**
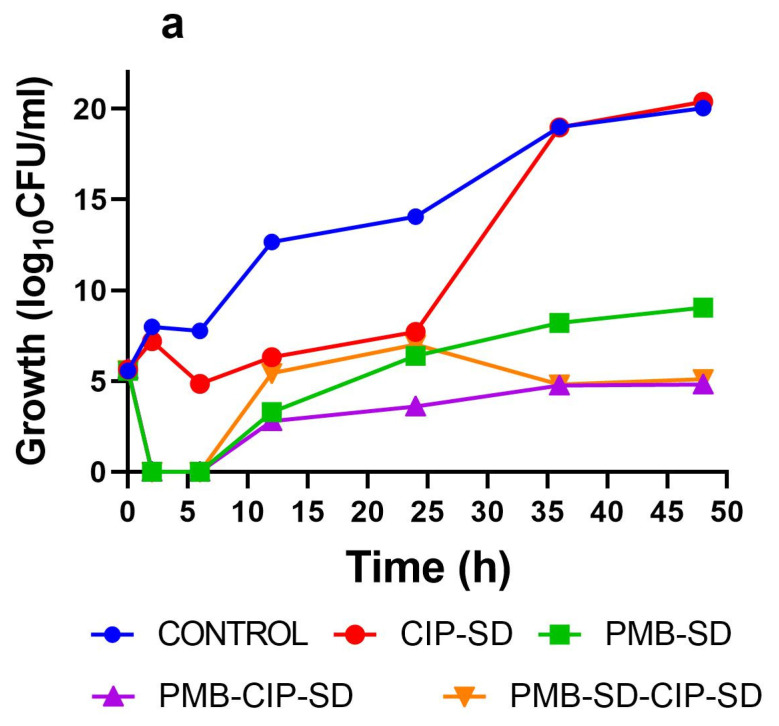

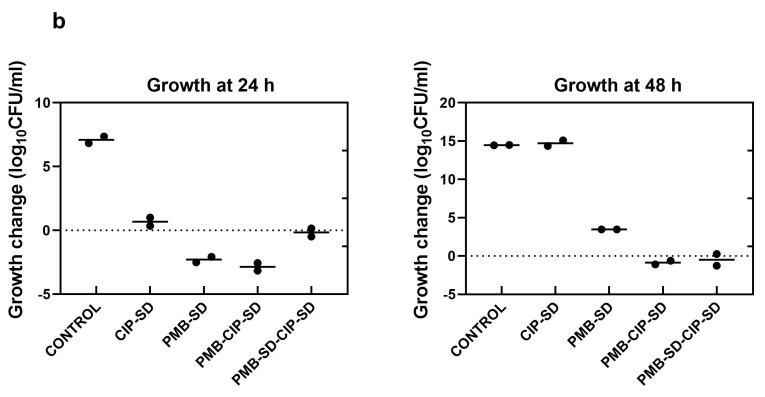
Time–kill assay with *A. baumannii* K31 treated with different formulations (**a**) and a histogram of the change in growth after 24 h and 48 h incubation compared to the inoculum (**b**). Data are presented as the means of two experiments.

The growth of bacteria treated with CIP–SD was inhibited for the first 24 h (0.7 log_10_ CFU/mL decrease as compared to the control, Figure 7), followed by regrowth from 24 h to 36 h, ultimately reaching a similar log_10_ CFU/mL to the control (i.e., 14.7 of log_10_ CFU/mL inoculum increase after 48 h of incubation). PMB–SD exhibited a bacteriostatic effect during the first 6 h, with a 5.6 log_10_ CFU/mL inoculum reduction. A regrowth could be seen after 6 h, but there was apparent inhibition compared to the control and CIP–SD–treated group (Figure 7). PMB–CIP–SD exhibited a similar bacteriostatic activity to the PMB group in the first 6 h, followed by regrowth. It also exhibited a stronger inhibition effect than the PMB group. As for PMB–SD–CIP–SD, a similar killing activity to that of PMB–SD could be observed at 0–24 h. However, the regrowth was slow between 24 and 48 h, and was similar to that of PMB–CIP–SD during the same period. The changes in log_10_ CFU/mL after 24 and 48 h incubation as compared to the inoculum are listed in Figure 7b. PMB–CIP–SD exhibited the strongest inhibition effect among the samples, followed by PMB–SD–CIP–SD, PMB–SD, and CIP–SD.

### 3.4. Population Analysis Profile

**Figure 8:**
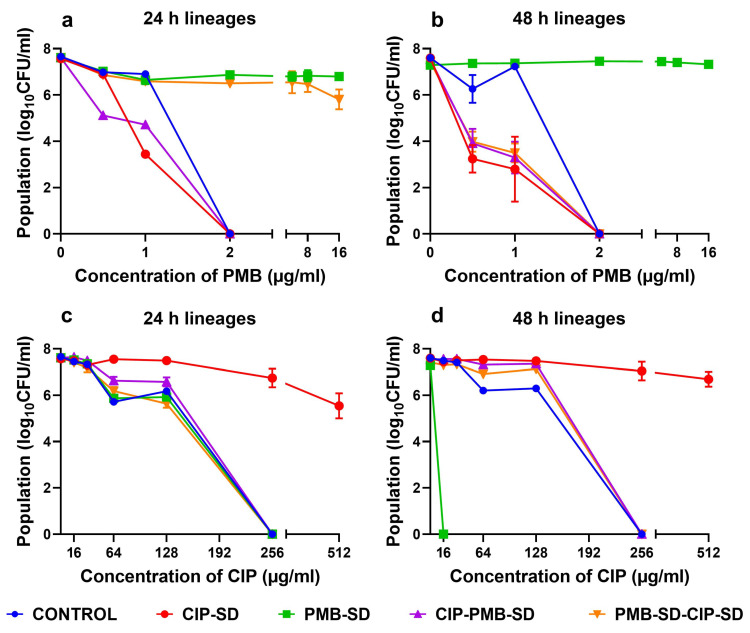
Population analysis profiles of lineages against PMB and CIP (mean ± SEM, n = 3). Lineages were treated with different antibiotics and have been coded by the names in the experimental design of the time–kill assay (Figure 1 and Table 3). (**a**,**c**) The results of lineages (after 24 h treatment in the time–kill assay) against different concentrations of PMB and CIP in the population analysis respectively; (**b**,**d**) the results of lineages (after 48 h treatment in the time–kill assay) against different concentrations of PMB and CIP in the population analyses, respectively.

In the population analysis of PMB using different concentrations, the PMB–SD 24 h and 48 h lineages were shown to be a resistant subpopulation that survived up to 16 µg/mL of PMB (Figure 8a,b). No resistant subpopulations were observed in the CIP–SD lineages (24 h and 48 h). the CIP–SD lineages became susceptible to lower concentrations of PMB compared to the control lineages. As shown in Figure 8a,b, reduced populations of the CIP–SD 24 h lineage and 48 h lineage can be observed at 1 µg/mL and 0.5 µg/mL of PMB, respectively. As for the drug combinations, PMB–CIP–SD was similar to CIP–SD. However, the PMB–SD–CIP–SD 24 h lineage was similar to the PMB–SD 24 h lineage, while the PMB–SD–CIP–SD 48 h lineage was similar to the CIP–SD 48 h lineage.

When the different lineages were exposed to varying concentrations of CIP, the CIP–SD lineages (24 h and 48 h) appeared to survive 512 µg/mL of CIP (Figure 8c,d). The PMB–SD 24 h lineage, PMB–CIP–SD 24 h lineage, PMB–CIP–SD 48 h lineage, PMB–SD–CIP–SD 24 h lineage, and PMB–SD–CIP–SD 48 h lineage were similar to the control lineages, i.e., no growth was seen at 256 µg/mL of CIP. Interestingly, the PMB–SD 48 h lineages were eliminate at 16 µg/mL of CIP (Figure 8d), which is much lower than the value of the control lineages (256 µg/mL).

### 3.5. Changes in MIC of Lineages

**Figure 9:**
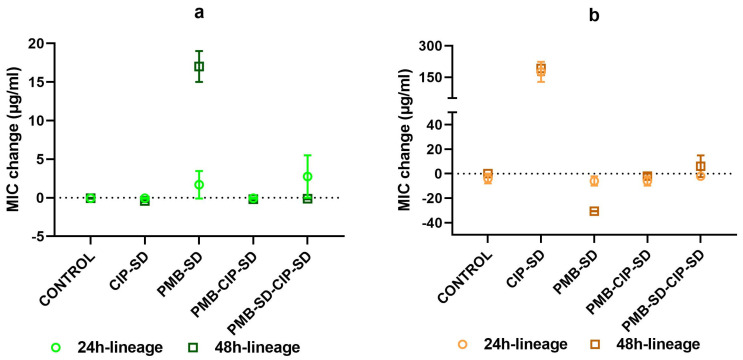
Change in MIC of PMB (**a**) and CIP (**b**) against lineages isolated from time–kill assay (mean ± SEM, n = 4). Lineages are grouped by names in the experimental design of the time–kill assay (Figure 1 and Table 3).

The change in MIC of PMB and CIP in various lineages isolated from the time–kill study are shown in Figure 9a,b, respectively. We observed a prominent increase in the MIC of PMB in the PMB–SD 48 h lineage, contrary to the MIC of the other lineages of PMB (Figure 9a). We also noted an increase in the MIC of CIP against the CIP–SD 24 h lineage and 48 h lineage, as well as a decrease in the CIP MIC for the PMB–SD 48 h lineage (Figure 9b). There was no change in the CIP MIC of other lineages.

### 3.6. Genomic Analyses

After purging repetitive and duplicated regions in the reference chromosome, we detected a total of 12 mutations in all sequenced isolates (n = 10) across ~3.2 Mb (80.39%) of the reference genome. All mutations were found in coding regions, with 59% (7/12) being non–synonymous (Supplementary Table S3).

## 4. Discussion

Spray–drying is a useful technology for preparing inhalable dry powders [33], with possibilities emerging of formulating drug combinations by the co–spray–drying of two or multiple active pharmaceutical ingredients (API) [34,35]. CIP is a poorly water–soluble fluoroquinolone antibiotic, and PMB is a water–soluble antibiotic. To load these two antibiotics with different solubilities, CIP was wet–milled to a homogeneous nano–suspension (Table S1 in Supplementary materials), and then mixed with PMB solution at a designated mass ratio prior to spray drying. The obtained PMB–CIP–SD exhibited a similar size distribution (Table 4) to CIP–SD and PMB–SD. According to the DVS results, CIP–SD showed abrupt sorption from 0 to 30% RH, followed by relatively gentle sorption from 30 to 70%, which indicates that CIP was able to form a hydrate when in contact with water [36]. The XRPD analyses suggest that the raw CIP was an anhydrate, and a CIP 3.7 hydrate was obtained in the spray–dried powders [37,38], i.e., CIP–SD and PMB–CIP–SD. This can be attributed to the interaction between water molecules and the CIP lattice during the wet ball–milling process, resulting in CIP hydrate nanoparticles [37]. This suggests that the conditions of the spray–drying process used in this study did not remove the water bound in the CIP nanoparticles.

There are no differences in the dissolution rates of PMB in different spray–dried powders (i.e., PMB–SD and PMB–CIP–SD). Similar dissolution rates of CIP were also observed in different spray–dried powders (i.e., CIP–SD and PMB–CIP–SD). The dissolution rate of the CIP derived from the spray–dried powders was higher than that of the raw CIP material (Figure 5). One possible reason is that the sizes of CIP in the spray–dried powders were smaller than those in raw CIP materials [39]. In addition, their solid forms were different (Figure 3). It is apparent that the dissolution rates of PMB were faster than those of the CIP from the dry powders in all formulations. This can be attributed to the differences in the intrinsic dissolution rates of PMB and CIP 3.7 hydrate.

In general, all spray–dried powders used in this study are inhalable, since a FPF value above 30% is an index of robust aerosol performance [16]. Notably, PMB–SD possesses a significantly higher respirable fraction compared to CIP–SD (Figure 6), while they have similar geometric particle sizes and size distributions (Table 4). The respirable faction of PMB–CIP–SD was around 60%. The inclusion of PMB in the spray–dried formulations seemed to improve the aerosol performance of CIP formulations. Studies on the spray–drying of PMB are rare, while spray–drying colistin (polymyxin E), its congener, has been more intensively studied. Colistin is known to improve the aerosol performance of co–spray–dried powders by inhibiting the cohesiveness of spray–dried particles [40,41].

As shown in Figure 6, the antibacterial effect of the combination of PMB–CIP–SD against *A. baumannii* K31 is more effective than those of either antibiotic used alone (i.e., CIP–SD and PMB–SD). The reduction in the log_10_ CFU/mL of PMB–CIP–SD was more than 2–fold at both 24 and 48 h, indicating the combination exerted a synergistic effect [42]. This indicates that the combination of PMB and CIP may be a promising candidate to treat infections caused by resistant *A. baumannii*, owing to their synergistic effects. While antibiotic combinations with synergistic effects eliminate bacteria rapidly, they may also create a window for mutant populations to develop and proliferate, resulting in an increase in resistance development [19,20]. Therefore, in the subsequent experiments, various lineages collected from the time–kill study were investigated by PAP analysis and MIC testing so as to gain further insights into the resistance development and collateral sensitivity of the drug combination.

The lineages collected at 48 h in the time–kill study with individual antibiotics (i.e., CIP–SD or PMB–SD) exhibited resistance. Interestingly, though, the PMB–SD lineages exhibited susceptibility to CIP. The CIP MIC decreased from 32 to 4.7 µg/mL. In addition, as shown in PAP, the resistant subpopulation was reduced in the presence of CIP, which could be considered as indicating collateral sensitivity [43,44].

The lineages treated by the combinations (i.e., PMB–CIP–SD and PMB–SD–CIP–SD) exhibited slower resistance development as compared to strains exposed to the individual antibiotics alone. The similar PAP and unchanged MICs seen in the PMB and CIP were similar to the findings for the control (i.e., bacteria that were not exposed to antibiotics). One possible reason is that the combination rapidly and more effective eradicated the bacteria than the individual antibiotics alone, limiting the time window of regrowth of the resistant mutants [20]. Another reason could be that the presence of PMB induced collateral sensitivity in the CIP. It has been reported that the development of resistance to an antibiotic combination could be limited when the resistance to one antibiotic confers collateral sensitivity to the other antibiotic [22].

The only difference between the two combined formulations, i.e., PMB–CIP–SD and PMB–SD–CIP–SD, is the sequence of the addition of CIP–SD in the time–kill study. The intention of testing PMB–SD–CIP–SD was to investigate whether the sequential use of antibiotics (PMB–SD first, followed by CIP–SD) afforded a better bactericidal effect and the greater inhibition of resistance development than the fixed–dose combination (PMB–CIP–SD). In addition, this will shorten the exposure time of CIP, and can take advantage of the collateral sensitivity of PMB. The results show that even though CIP–SD was added to the bacterial culture 24 h after the treatment of PMB (i.e., PMB–SD–CIP–SD), the antibacterial effects of the two formulations at 48 h were similar (Figure 7, Figure 8 and Figure 9). The possible reason for this could be that the change in the population with collateral sensitivity (treated with PMB solution) within 24 h was not high enough to induce the antibacterial activity (i.e., time–kill, PAP and MIC). The mutant frequencies were estimated, and 59% of identified mutants were found to be non–synonymous. This suggests that the single–drug–treated lineages collected in this study adapted to the antibiotics (CIP and PMB) without mutation [44]. Consequently, the collateral susceptibility observed in this study can be attributed to the pre–adaptation phenomenon, which has been found to be associated with beta–lactamase and efflux pump activities [45,46].

Contemporary antibiotic treatments via the oral route and injection cannot always reach an adequate bacteria–killing effect for chronic respiratory infection [47]. The optimization of the exposure–response relationships of antibiotics is beneficial to the treatment of severe infections in the lung, which can be performed via the pulmonary administration of antibiotics [47]. The pulmonary administration of antibiotics such as tobramycin and colistin was first undertaken using nebulized solutions [48]. Inhaled tobramycin, i.e., TOBI^®^ Podhaler™, was approved first in 2013 by the FDA as an inhaled antibiotic dry powder product, bringing obvious clinical benefits to respiratory infection treatment [49]. The need for dry powder inhalers is increasing rapidly since they are portable and convenient for use [50]. Moreover, the development of new inhaled antibiotic combinations has not stopped. For example, besides the antibiotic pairs that afford synergistic effects [51,52], new combinations, such as antibiotic–biologicals, are also being studied in inhalable dry powder forms [53]. In addition, new delivery systems, such as nanoparticles and liposomes aiming to overcome the mucus/sputum barrier and prolong the drug retention time in the lung of inhaled antibiotics for the treatment of chronic respiratory infection, have been investigated and developed, as these delivery systems could provide additional functionality to the treatment [52,54].

In brief, resistance development is an important factor that should be considered in rational combination designs, and collateral sensitivity/resistance studies may offer more opportunities, and inspire the development of new resistance–limiting combinations.

## 5. Conclusions

This study demonstrates that inhalable dry powders consisting of CIP and PMB can be readily produced by spray–drying. The fixed–dose combination of CIP and PMB is affordable and more effective against multidrug–resistant *A. baumannii*. In addition, this combination exerts a synergistic effect, and can better suppress the development of resistance as compared to individual antibiotics alone.

## Figures and Tables

**Table 1:** Minimum inhibitory concentrations (MICs) of different antibiotic against *A. baumannii* K31.

Antibiotics	MICs (µg/mL)	MIC Break Points (µg/mL)	
		S≤	R>
Aztreonam	64	N	N
Ciprofloxacin	32	0.25	0.5
Polymyxin B *	1	–	–
Tobramycin	256	4	4

S: susceptible; R: resistant; according to European Committee on Antimicrobial Susceptibility Testing (EUCAST) [27]. N: No breakpoints, and susceptibility testing is not recommended as the bacterial species is a poor target for therapy with this antibiotic (isolates may be reported as R without prior testing). * No breakpoints listed for polymyxin B, but colistin breakpoints are S ≤ 2 and R > 2 (PMB and colistin both belong to polymyxins group).

**Table 2:** Feeding solutions for spray–drying.

Dry Powders	Abbreviation	Solid Contents of Feeding Solution	
		PMB Solution (mg/mL)	CIP Suspension (mg/mL)
PMB spray–dried powder	PMB–SD	9.6	–
CIP spray–dried powder	CIP–SD	–	9.8
PMB–CIP co–spray–dried powder	PMB–CIP–SD	0.31	9.8

The size distribution of nanoparticles in CIP suspension was measured by dynamic light scattering, see detailed description in Supplementary Materials.

**Table 3:** Group setting and corresponding antibiotic addition plan.

Code	Name	Treatment	Concentration (µg/mL)	
			CIP	PMB
a	Control	no antibiotic	–	–
b	PMB–SD	add PMB–SD at time zero	–	1
c	CIP–SD	add CIP–SD at time zero	32	–
d	PMB–CIP–SD	add PMB–SD and CIP–SD at time zero	32	1
e	PMB–SD–CIP–SD	add PMB–SD at the beginning then add CIP–SD added after 24 h	32	1

Concentrations of CIP and PMB tested corresponded to pure drug formulations.

**Table 4:** Particle sizes of dry powder formulations.

Sample Name	Diameter (µm)			Span (µm)
	Dv_10_	Dv_50_	Dv_90_	
PMB–SD	1.2 ± 0.1	2.7 ± 0.1	5.7 ± 0.3	1.6 ± 0.1
CIP–SD	0.7 ± 0.1	2.6 ± 0.2	6.4 ± 0.3	2.2 ± 0.1
PMB–CIP SD	0.7 ± 0.1	2.7 ± 0.1	6.3 ± 0.1	2.0 ± 0.1

Dv_10_, Dv_50_, and Dv_90_ are volumetric diameters at 10%, 50% and 90% cumulative numbers, respectively. Data shown are representative of triplicate tests (mean ± SD, n = 3).

## Data Availability

Data are available on request.

## Supplementary Materials

The following supporting information can be downloaded at: https://www.mdpi.com/article/10.3390/pharmaceutics15030720/s1, Figure S1: DVS isotherms for PMB–SD, CIP–SD and PMB–CIP–SD; Figure S2: Time–kill assay result of *A. baumannii* K31 treated with different solutions; Figure S3: Population analysis profiles of lineages used against PMB and CIP; Figure S4: Changes in MIC of PMB and CIP against lineages isolated from time–kill assay. Table S1: Particle size of micronized CIP nano–suspension; Table S2: Group setting and corresponding antibiotic supplementation plan; Table S3: Genetic differences between isolates from different experimental groups with the reference of *A. baumannii* 1656–2.
