# Supplementary material for: Drug Combination of Ciprofloxacin and Polymyxin B for the Treatment of Multidrug–Resistant Acinetobacter baumannii Infections: A Drug Pair Limiting the Development of Resistance
Source: Pharmaceutics. 2023 Feb 21;15(3):720. doi: 10.3390/pharmaceutics15030720 (PMC10056848; doi:10.3390/pharmaceutics15030720)
Supplement: Supplementary file 1 [file pharmaceutics-15-00720-s001.zip › Supplymentary materials (DVS_PC-solutions_genom).pdf]

Supplementary materials

Dynamic light scattering

Method

The intensity-weighted mean hydrodynamic particle diameters (z-average) of ciprofloxacin (CIP) nano-suspension were determined by dynamic light scattering (DLS) (Malvern Nano ZS, Malvern Instruments, Malvern, UK). The suspension was sonicated for 3 min at room temperature, and 1 mL suspension (CIP concentration 0.5 mg/mL) was added in the cuvette for the measurement.

Result

Table S1. Particle size of micronized CIP nano-suspension (Mean ± SD, n = 3)

	**Particle size**	**Size distribution**
CIP nano-suspension	362 ± 10 nm	0.21 ± 0.1 (PDI)

Polydispersity index (PDI) was measured for nanocrystal suspension to perform size distribution.

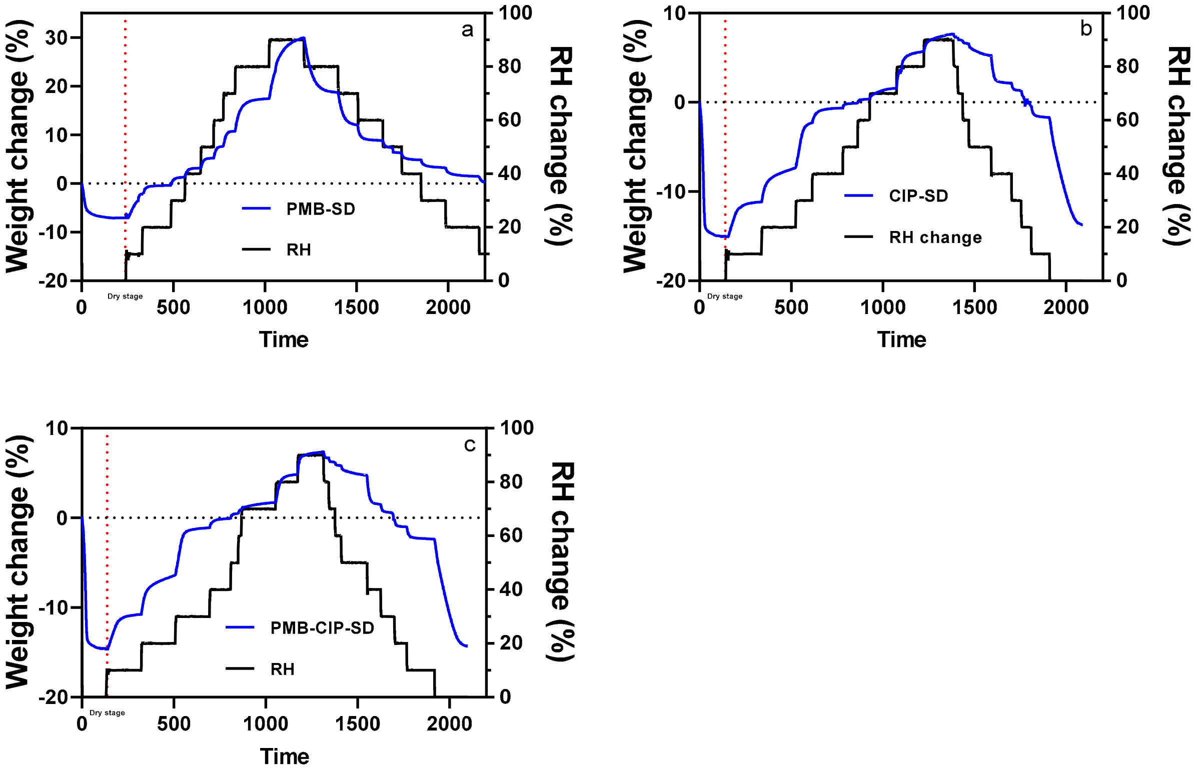

Figure S1. DVS isotherms (weight change-time) for PMB-SD (a), CIP-SD (b) and PMB-CIP-SD (c). The vertical dotted lines represent the drying process of DVS measurements, at 60°C, RH 0%. The description of the method can be found in the main

Design of in-vitro *microbiology study of solutions (parallel with dry powders in main body of the article)*

Solutions with free antibiotics were employed as a reference (*i.e.* time-kill assays, population analysis, and MIC test), and the design was the same as the test performed on antibiotic dry powders. The only difference here is the use of antibiotic solutions instead of spray dried powders. The detailed description of each test can be found in the main body of the article.

Results

Table S2. Group setting and corresponding antibiotic adding plan. The experimental design are same with the dry power groups in main body (Figure 1).

Code	Name	Abbreviation	Dosing regimen	Concentration (μg/mL)	
				CIP	PMB
f	Control	NA	No antibiotic adding	NA	NA
g	PMB solution	PMB-Sol	treated with free PMB singly at 1 μg/mL	NA	1
h	CIP solution	CIP-Sol	treated with free CIP singly at 32 μg/mL	32	NA
i	PMB-CIP combination solution	PMB-CIP-Sol	treated with free PMB (1 μg/mL) and CIP (32 μg/mL)	32	1
j	Sequential addition of PMB solution and then CIP solution	PMB-Sol-CIP-Sol	bacteria was treated with free PMB (1 μg/mL) at beginning then CIP (32 μg/mL) after 24 h	32	1

NA: not applicable to this sample.

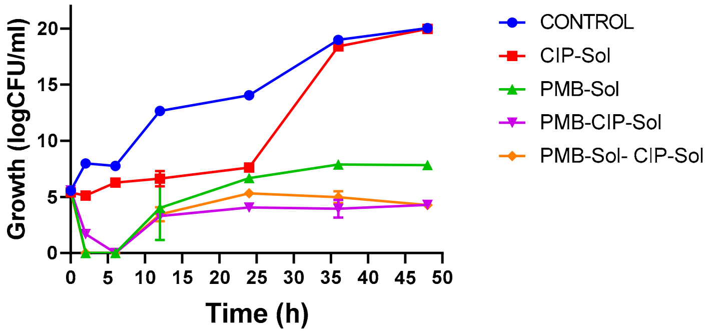

Figure S2. Time-kill assay result of *A. baumannii* K31 treated with different solutions. Data are presented as the means of two experiments.

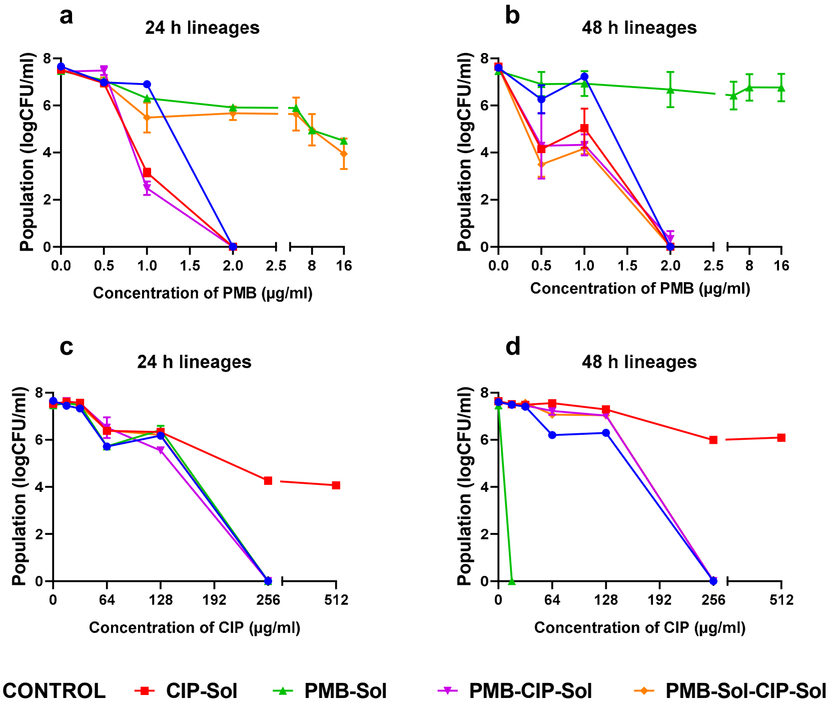

Figure S3. Population analysis profiles of lineages against PMB and CIP (mean ± SEM, n = 3). Lineages were treated by different antibiotics and coded by the names in the experiment design of the time-kill assay (Figure 1 and Table 3). a, c: the results of lineages after 24 h treatment in the time-kill assay, against different concentrations of PMB and CIP in population analysis respectively; b, d: the results of lineages after 48 h treatment in the time-kill assay, against different concentrations of PMB

and CIP in population analysis respectively.

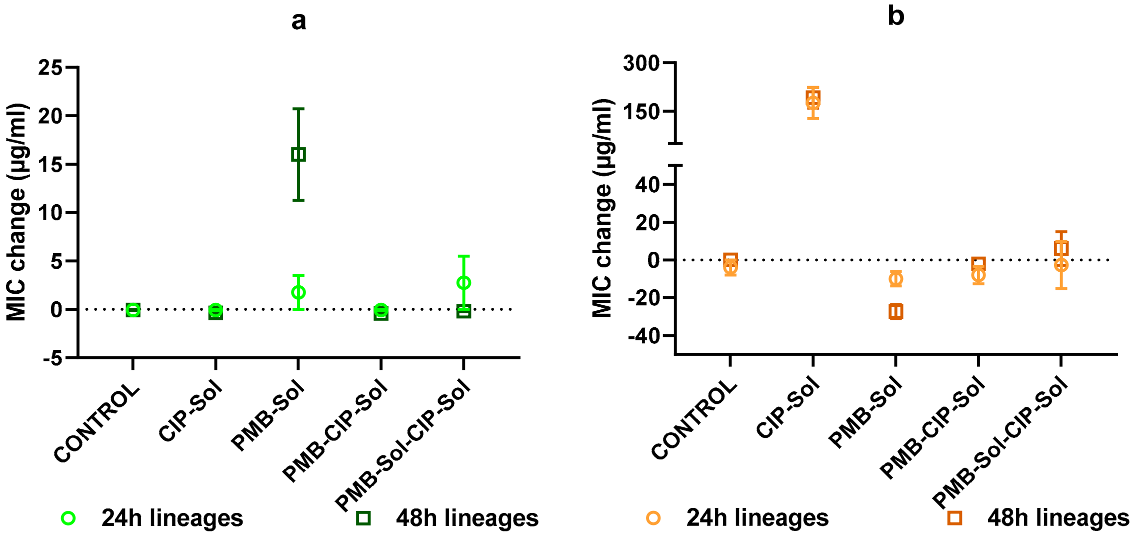

Figure S4. Change in MIC of PMB (a) and CIP (b) against lineages isolated from time-kill assay (mean ± SEM, n = 4). Lineages were grouped by the names in the experiment design of the time-kill assay (Table S2).

Genomic analyses

After the time-kill study, *A. baumannii* isolates from the control, CIP-Sol, and PMB-Sol experiments were collected for genomic analyses. The raw sequence data was aligned to the annotated *A. baumannii* 1656-2 reference chromosome and detected single nucleotide polymorphisms arelisted in Table S3. For Control, CIP-Sol, and PMB-Sol lineages, one isolate was selected randomly for each; for PMB-CIP-Sol and PMB-Sol-CIP-Sol lineages, three isolates were selected randomly for each.

Table S3. Genetic differences between isolates from different experiment groups with a reference of *A. baumannii* 1656-2

	A. baumannii K31	CIP-Sol-1	PMB-CIP-Sol-1	PMB-CIP-Sol-2	PMB-CIP-Sol-3	Control	PMB-Sol	PMB-Sol-CIP-Sol-1	PMB-Sol-CIP-Sol-2	PMB-Sol-CIP-Sol-3
A. baumannii K31		6	3	3	3	3	3	3	5	6
CIP-Sol-1	6		3	3	5	3	3	3	5	6
PMB-CIP-Sol-1	3	3		0	2	0	0	0	2	3
PMB-CIP-Sol-2	3	3	0		2	0	0	0	2	3
PMB-CIP-Sol-3	3	5	2	2		2	2	2	4	3
Control	3	3	0	0	2		0	0	2	3
PMB-Sol	3	3	0	0	2	0		0	2	3
PMB-Sol-CIP-Sol-1	3	3	0	0	2	0	0		2	3
PMB-Sol-CIP-Sol-2	5	5	2	2	4	2	2	2		5
PMB-Sol-CIP-Sol-3	6	6	3	3	5	3	3	3	5	
